# Modeling and prediction of conduction delay in an unmyelinated axon

**DOI:** 10.1186/1471-2202-13-S1-P81

**Published:** 2012-07-16

**Authors:** Yang Zhang, Dirk Bucher, Farzan Nadim

**Affiliations:** 1 Dept Math Sci and Dept Biol Sci, New Jersey Institute of Technology, Newark, NJ, 07102, USA; 2 The Whitney Lab for Marine Bioscience and Dept of Neuroscience, University of Florida, St. Augustine, FL, 32080, USA

## 

Conduction delay in axons is a function of the axon morphology, passive membrane properties and voltage-gated ionic currents. Although conduction delay is usually assumed to be constant, indicating perfect temporal fidelity, recent work has shown that the delay of each action potential depends on the prior short- and long-term history of activity in the axon [1]. Violation of temporal fidelity leads to substantial variations in inter-spike intervals which has potential impact on temporal neural activity.

In experiments using the motor axon of the pyloric dilator (PD) neuron of the lobster, *H. americanus*, we measured conduction delay using Poisson random stimuli applied at different mean rates [2]. These measurements showed that the mean value and the variance of conduction delay increases as a function of stimulus time until these values reach a steady state at about 5 minutes post-stimulation. Even at steady state, conduction delay showed a non-monotonic relationship with stimulus frequency (f_inst_): it was higher at small or large values of f_inst_ but has a minimum at f_inst_~45 Hz. Moreover, bath application of dopamine drastically improved the temporal fidelity of conduction delay.

We have constructed a conductance-based model of the PD axon to examine the role of different ionic currents in shaping the history dependence of conduction delay. In addition to the standard Hodgkin-Huxley currents, this model includes a transient potassium current I_A_ and a hyperpolarization activated inward current I_h_, both of which have been characterized in this axon. The main effect of dopamine in the biological axon is an increase of I_h_ which counteracts the activity-dependent hyperpolarization [3], mimicked by adding a Na^+^/K^+^ pump in the model.

**Figure 1:**
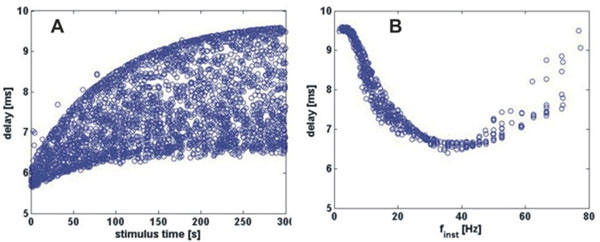
Changes in action potential delay in the model axon in response to 5 mins of Poisson stimulation (mean rate 10 Hz). Compare with experimental data [2]. **A.** Both mean and CV of conduction delay increase slowly in response to activity. **B.** Delay (fifth minute) shows a non-monotonic relationship with stimulus frequency.

We find that the Na^+^/K^+^ pump enables the model to reproduce the long-term history dependence observed in the biological PD axon. A pump current with a slow time constant (τ=90 sec) results in a slow increase of the mean and CV of conduction delay with stimulus time (Fig. 1A), and a non-monotonic relationship between conduction delay and f_inst_ (Fig. 1B), as observed experimentally [2]. Additionally, as in the experiments, temporal fidelity is improved as I_h_ is increased.

Action potential conduction velocity can be approximated by  (*d*=axon diam, R^*^=total memb resistance/unit area at peak, *R_a_*=axial resistivity, *C_m_*=capacitance/unit area [4]. Although this equation accurately predicts the conduction velocity of a single action potential in our model, it fails to the variations in conduction velocity in the presence of the history-dependent effects. This discrepancy is partly due to non-uniform conduction velocity along the axon when active at high rates. We revise the formula to predict the relationship between the conduction delay and total membrane resistance at the action potential peak.
